# Andrographolide derivative AL-1 ameliorates TNBS-induced colitis in mice: involvement of NF-кB and PPAR-γ signaling pathways

**DOI:** 10.1038/srep29716

**Published:** 2016-07-20

**Authors:** Yali Yang, Hui Yan, Mei Jing, Zaijun Zhang, Gaoxiao Zhang, Yewei Sun, Luchen Shan, Pei Yu, Yuqiang Wang, Lipeng Xu

**Affiliations:** 1 Institute of New Drug Research and Guangzhou Key Laboratory of Innovative Chemical Drug Research in Cardio-cerebrovascular Diseases, Jinan University College of Pharmacy, Guangzhou 510632, China

## Abstract

Andrographolide is a traditional herb medicine, widely used in Asia for conditions involving inflammation. The andrographlide-lipoic acid conjugate, AL-1, has been found being able to alleviate inflammation in our previous reports. Although the anti-inflammatory activity of AL-1 contributes to its cytoprotective effects, whether AL-1 can improve inflammatory bowel disease (IBD) and the underlying mechanisms of its action remain largely unknown. In this study, we investigated the anti-inflammatory effects of AL-1 in C57BL/6 mice with trinitrobenzenesulfonic acid (TNBS)-induced colitis. The body weight loss and length change of colon after TNBS instillation were more severe than those in normal mice. AL-1 treatment led to significant reductions in disease activity index (DAI), macroscopic score and colon mucosa damage index (CMDI) associated with TNBS administration. AL-1 inhibited the inflammatory response via lowering the level of inflammatory cytokines and myeloperoxidase (MPO) activity. AL-1 attenuated the expression of p-p65, p-IκBα and COX-2 in the colitis mice. The alleviation of colon injury by AL-1 treatment was also evidenced by the increased expression of PPAR-γ. These results indicated that AL-1 could protect intestinal tract from the injury induced by TNBS in mice, suggesting that AL-1 may have potential in treatment for IBD.

Inflammatory bowel disease (IBD), which consists of ulcerative colitis and Crohn’s disease, refers to immunologically mediated inflammatory disorder of the gastrointestinal tract^1^. IBD afflicts nearly 1.5 million Americans and 2.2 million people in Europe and several hundred thousands more worldwide^2^,^3^. However, the precise pathogenesis of IBD is not well understood. There is growing evidence that the delicate balance among the microbiota, the intestinal epithelium and the immune system sustains the health of gastrointestinal tracts^4^. Once the homeostasis breaks down and shifts to the pro-inflammatory side, hyperactive immune cells secrete the pro-inflammatory cytokines including TNF-α, IL-1β and IL-6 through the activation of regulatory mechanisms such as the NF-κB and PPAR-γ pathways^5^,^6^,^7^. These signaling cascades would increase people’s susceptibility to IBD and eventually precipitate the chronic inflammatory pathology found in the disease. NF-κB, comprising the p65 and p50 subunits, plays a crucial role in controlling inflammatory response of immune disease^8^. Therefore, blockade of NF-κB activation would be a robust therapeutic intervention for IBD. Likewise, the activation of PPAR-γ would alleviate the inflammatory processes of IBD^7^.

There are intense demands of more optimal medical therapies for IBD accompanying with the understanding of the pathogenesis of IBD. In recent years, there are extensive research on using herbal medicines as potential agents for IBD^9^,^10^,^11^. *A. paniculate* leaves are rich in the andrographolide and are widely employed in folk medicine as antibacterial, anti-asthmatic, antiviral, and neuroprotective and anti-inflammatory agents^12^,^13^,^14^,^15^. Andrographolide sulfonate, approved as an anti-inflammatory drug in China for year, has showed significant activity in TNBS-induced colitis in mice^16^. Likewise, alpha-lipoic acid has been identified as a potential remedy of IBD^17^,^18^,^19^. AL-1 (Fig. 1), an andrographolide-lipoic acid conjugate, has shown anti-inflammatory effects in our previous studies^15^,^20^. In this study, we investigated the therapeutic effects and mechanisms of AL-1 in TNBS-induced colitis in mice.

## Results

### The anti-inflammatory effects of AL-1 in mice with TNBS-induced colitis

**Figure 2:**
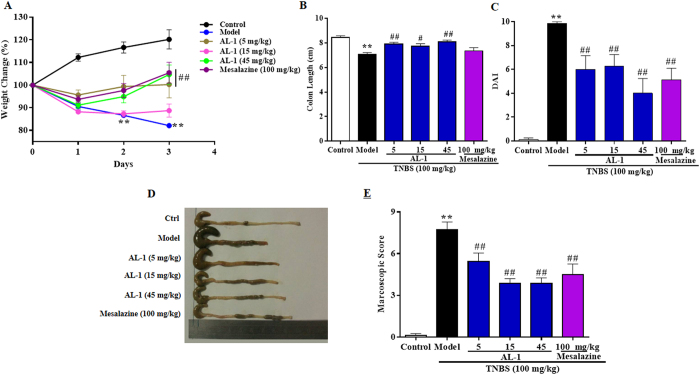
Effects of AL-1 on colitis induced by TNBS instillation in C57BL/6. (**A**) The time-course of body weight changes on day 3 after TNBS-induced colitis. (**B**) Effects of AL-1 and mesalazine on colon length of TNBS-induced colitis mice. (**C**) Disease activity index calculated as described in material and methods. (**D**) Representative photograph of colons from day 3 after the induction of TNBS-colitis. (**E**) Macroscopic score. Mice were challenged with solvent or TNBS at day 0 and treated with control, AL-1 (5, 15 and 45 mg/kg) or mesalazine (100 mg/kg) for days 0-3. Body weight was measured daily throughout the experiment. Values were shown as the means ± SEM, n = 8 for each group. **P < 0.01 vs control group, ^*#*^*P *< 0.05 and ^*##*^*P *< 0.01 vs mice treated with TNBS alone.

In order to examine whether AL-1 could improve the clinical symptoms of TNBS-induced colitis in mice, the clinical signs including weight changes, colon length, DAI score and macroscopic score were assessed. The significant weight loss, DAI score and macroscopic score and shortening colon length in model group manifested that colon instillation of TNBS resulted in a reproducible colitis in mice (Fig. 2A–F). As shown in Fig. 2B, AL-1 or mesalazine administration attenuated the declining of body weight which was observed in TNBS challenged mice. Treatment with AL-1 produced a significant improvement in colon length, DAI score and macroscopic score compared with those in the model group (Fig. 2B–E). Therefore, these data suggested that AL-1 ameliorated the severity of TNBS-induced injury in mice.

### AL-1 diminished colonic histopathological changes

**Figure 3:**
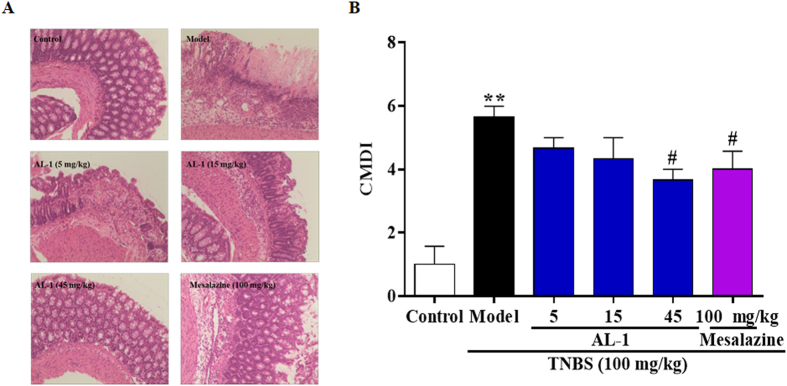
Effects of AL-1 on colonic histopathological changes. (**A**) Histological appearance of mice colonic mucosa after haematoxylin and eosin (H&E) stain (original magnification 100×). (**B**) The score of colon mucosa damage index (CMDI) of mice. Mice were challenged with solvent or TNBS at day 0 and treated with control, AL-1 (5, 15 and 45 mg/kg) or mesalazine (100 mg/kg) for days 0–3. After sacrificed at day 3, sections of the colon were collected, embedded in paraffin, and cut into 4 μm of sections and stained with hematoxylin and eosin before examination. Values were shown as the means ± SEM, n = 8 for each group. **P < 0.01 vs control group, *##P* < 0.05 vs mice treated with TNBS alone.

Based on the previous data, we then investigated whether AL-1 could alter histopathological damage in colons of mice with TNBS-induced colitis. The colon tissue from model group revealed typical characteristics of abnormal structure including loss of epithelial and goblet cells, crypt lesions and prominent transmural inflammatory cells infiltration in the intestine mucosa and submucosa (Fig. 3A). The degree of colitis was quantitatively evaluated using the scoring system described in materials and methods (Fig. 3B). Moreover, AL-1 improved the histopathological changes caused by TNBS.

### AL-1 suppressed recruitment of immune-inflammatory cells

**Figure 4:**
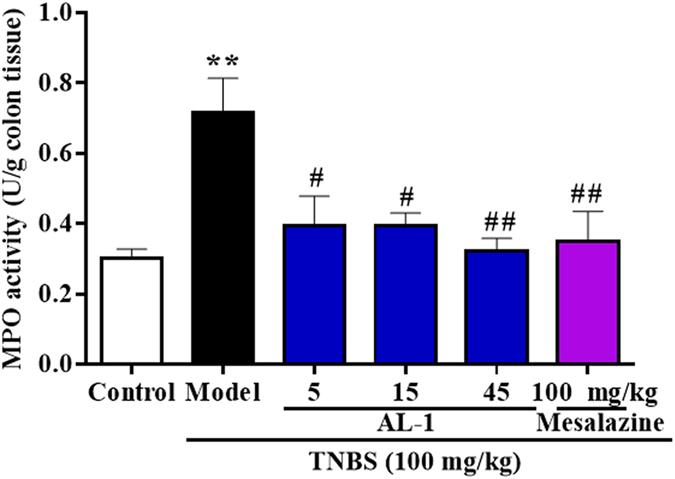
Effects of AL-1 on myeloperoxidase (MPO) activity in colonic tissues of mice. Mice were challenged with solvent or TNBS at day 0 and treated with control, AL-1 (5, 15 and 45 mg/kg) or mesalazine (100 mg/kg) for days 0–3. Protein from colon tissue was extracted and MPO level was determined according to manufacturer’s instructions. Values were shown as the means ± SEM, n = 8 for each group. **P < 0.01 vs control group, ^*#*^*P* < 0.05 and ^*##*^*P* < 0.01 vs mice treated with TNBS alone.

MPO activity, a biochemical maker for neutrophil influx, was assessed to detect the potency of AL-1 of resisting inflammatory cell infiltration. The alteration of MPO activity was consistent with the changes in histological score described before indicating that AL-1 drastically inhibited the pronounced damage induced by TNBS (Fig. 4).

### Secretion of pro-inflammatory cytokines was down-modulated by AL-1

**Figure 5:**
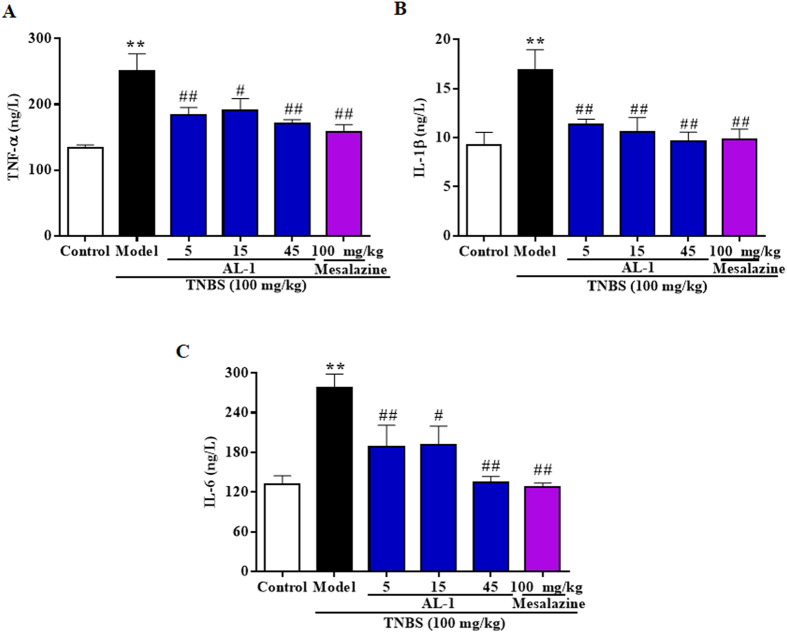
Inhibitory effects of AL-1 on the pro-inflammatory cytokine production in serum level of mice. The production of (**A**) TNF-α, (**B**) IL-1β and (**C**) IL-6, were assayed in colonic tissues of mice. Mice were challenged with solvent or TNBS at day 0 and treated with control, AL-1 (5, 15 and 45 mg/kg) or mesalazine (100 mg/kg) for days 0–3. The TNF-α, IL-1β and IL-6 concentration in the serum level were determined by ELISA. Values were shown as the means ± SEM, n = 8 for each group. **P < 0.01 vs control group, ^*#*^*P* < 0.05 and ^*##*^*P* < 0.01 vs mice treated with TNBS alone.

Next, we measured the effects of AL-1 on the expression of pro-inflammatory cytokines TNF-α, IL-1β and IL-6. As depicted in Fig. 5, TNBS instillation promoted the expression of TNF-α, IL-1β and IL-6 in mice. In contrast with the model group, AL-1 remarkably attenuated the levels of pro-inflammatory cytokines TNF-α, IL-1β and IL-6.

### AL-1 modulated the expression of COX-2, NF-κB and PPAR-γ

**Figure 6:**
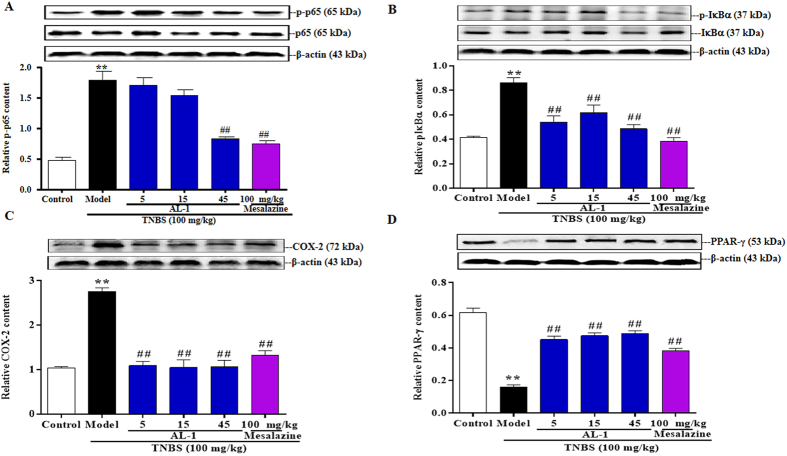
Effects of AL-1 on protein expression of COX-2, p-p65, p-IκBα and PPAR-γ in colon tissues. (**A**–**D**) Mice were challenged with saline or TNBS at day 0 and treated with control, AL-1 (5, 15 and 45 mg/kg) or mesalazine (100 mg/kg) for days 0–3. Values were shown as the means ± SEM, n = 8 for each group. **P < 0.01 vs control group, ^##^*P* < 0.01 vs mice treated with TNBS alone.

Furthermore, we investigated the possible role of AL-1 in the expression of COX-2, NF-κB and PPAR-γ in mice with TNBS colitis (Fig. 6). Mice in the model group exhibited a tendency to up-regulate COX-2, p-p65 and p-IκBα expression. However, an attenuated trend was observed in the groups treated with AL-1. AL-1 administration increased the expression of PPAR-γ which might act as an anti-inflammatory role in the progress of colitis (Fig. 6D). Together, the ability of AL-1 to modulate the expression of COX-2, NF-κB and PPAR-γ conferred anti-inflammatory capacity to AL-1 in IBD.

## Discussion

In this study, we demonstrated that AL-1 could alleviate the severity and extent of colonic damage caused by TNBS in mice. AL-1 acts through down-regulating NF-κB pathway and up-regulating PPAR-γ pathway to reduce the activity of MPO and the accumulation of inflammatory cytokines including TNF-α, IL-1β and IL-6. By targeting NF-κB pathway and PPAR-γ pathway, AL-1 could maintain the balance inflammatory and anti-inflammatory to preserve integrity of the gastrointestinal tract.

Intracellular MPO has prominent effects in destruction of microorganisms, however, its release in extracellur matix may lead to the damage of host tissues with inflammation^21^,^22^. MPO is a well-established biomarker of neutrophil infiltration for evaluating inflammation in humans and animal model of IBD^23^,^24^,^25^,^26^. In our study, AL-1 significantly inhibited the increased MPO activity induced by TNBS in mice. Likewise, histopathological changes which represent the crypt loss and substantial leukocyte infiltration, showed a profile similar to the change of MPO activity in different groups.

Recent genetic and immunological studies have shown that the involvement of cytokines (TNF-α, IL-1β and IL-6) contributed to IBD perpetuation and tissue destruction^27^,^28^,^29^. Therefore, that anti-cytokine therapies involving anti-TNF-α agent is now commonly used for IBD in the clinic indicates cytokine inhibitor will be a significant field of interest for IBD therapy^30^. Therefore, anti-cytokine prescription might pave the way to more effective clinical approaches that targets multi-cytokines blocker that could suppress several cytokines simultaneously^29^. Our data indicated that AL-1 could simultaneously inhibit the expression of TNF-α, IL-1β and IL-6.

The overwhelming activation of NF-κB would aggravate the severity of intestinal inflammation in IBD patients^31^. Therefore blockade of NF-κB activation became a promising therapeutic strategy in IBD^31^,^32^. The phosphorylation of p65 and IκBα which initiate the activation of NF-κB are the key regulators in the NF-κB pathway. In this regard, we wondered whether AL-1 inhibited NF-κB activation to exert its protective effect in TNBS induced mice colitis. We discovered that AL-1 markedly down-regulated the expression of p-p65 and p-IκB proteins in the experimental colitis. As anticipated, the elevation COX-2 which is regulated by NF-κB in colitis mice could be restored by AL-1 administration. There is a negative correlative between NF-κB and PPAR-γ which is an upstream target of NF-κB^33^,^34^. In our study, the expression of PPAR-γ was markedly increased in AL-1 treated groups. These results suggested that the effect of AL-1 protecting mice from TNBS-induced colonic injury was very likely mediated by activation of PPAR-γ.

In summary, morphometric and histological indices of colitis revealed the protective effect of AL-1 in TNBS induced colitis. Furthermore, AL-1 could ameliorate the experimental colitis by regulating NF-κB and PPAR-γ signaling pathway. Our study demonstrated that AL-1 might be a promising therapeutic agent of IBD in future.

## Materials and Methods

### Regents

AL-1 was previously synthesized and purified in our laboratory. Trinitrobenzenesulfonic acid (TNBS) was purchased from Sigma-Aldrich (St. Louis, MO). Kits for determining myeloperoxidase (MPO) and ELISA kits for TNF-α, IL-1β and IL-6 were purchased from Nanjing Jiancheng Bioengineering Institute (Nanjing, China). Antibodies against p65, p-p65, IκBα, p-IκBα, COX-2 and PPAR-γ were purchased from Cell Signaling Technology Inc (Boston, MA). Antibodies against p50 and β-actin were purchased from Abcam (Cambridge, MA, USA). All other reagents were obtained from Sigma Chemical Co. (St. Louis, MO).

### Animals

C57BL/6 (aged 6–8 weeks) were purchased from Guangdong Medical Laboratory Animal Center (Guangzhou, China). The mice were kept in the temperature controlled room with 12 h dark/light cycles, and were allowed free food and water ad libitum. All animal welfare and experimental procedures were approved by the Research Ethics Committee of Jinan University. In addition, animal experiments were performed in accordance with relevant guidelines and regulations.

### Induction of colitis and treatments

Colitis was induced in accordance with the method as described previously^35^. Briefly, animals were fasted for 18–24 hours with free access to 5% glucose solution. Subsequently, mice were anaesthetized by inhaling diethyl ether. Mice were randomly assigned into control and colitis groups. A medical grade catheter was gently inserted into colon (4 cm proximal to the anus). In order to induce colitis, TNBS (100 mg/kg, 50% ethanol solution) was slowly instilled into colon. A head down position was applied to ensure TNBS fully distributed in the entire colon for 3 min. The control group received 100 μl of 50% ethanol alone through the same technique. The animals were then given free access to food and water. Throughout the experiments, mice were monitored for body weight loss. At 72 hour following TNBS administration, the animals were killed, and the colon was removed, dissected and opened lengthwise.

To evaluate the therapeutic effect of AL-1 in experimental colitis, animals were administered orally with different doses of AL-1 twice a day (5, 15 and 45 mg/kg) 3 h before TNBS instillation. AL-1 was suspended in 5% polyvinyl alcohol 17–88 solution which contain 1% 1, 3-Propanediol, 1% Tween 80 and 1% ethanol. The same solvent was given to both the control and model groups. Mesalazine was used as a positive control and given to the animals with induced colitis at a dose of 100 mg/kg daily, i.g.

### Evaluation of macroscopic scores

Evaluation pattern for macroscopic characteristics was determined by previously established scoring system (Table 1)^36^.

### Evaluation of disease activity index (DAI)

Evaluation pattern for disease activity was determined by previously established scoring system (Table 2)^37^.

### Histopathological examination

Colon tissues were collected and fixed into 4% buffered formaldehyde solution overnight at 4 °C. The fixed tissues were cut into small sizes and put in a labelled tissue cassette for dehydration processing. The tissues were cleaned twice with xylene before being embedded in paraffin. Paraffin sections were cut into slices of 4 μm and stained with H&E staining solution. Finally, the stained sections were observed and photographed under a light microscope (with 100× magnification). Colon mucosa damage index scoring was assessed in a blinded fashion as described in Table 3^38^.

### Determination of inflammatory cytokine levels

Serum was collected from blood of mice by centrifuge at 3500 g for 15 min. Serum cytokine levels were measured by specific ELISA kits from Nanjing Jiancheng Bioengineering Institute.

### Myeloperoxidase (MPO) assay

Protein extracted from colonic tissue was used to determined MPO level according to manufacturer’s instructions. The results were expressed as activity units per mg tissue.

### Western blot assay

To investigate the mechanism of AL-1 improved colitis induced by TNBS, we examined the effect of AL-1 on the expression of COX-2, NF-κB and PPAR-γ by western blotting. Protein from colonic tissue was extracted in lysis buffer. The protein concentration was then measured using a BCA Protein Assay kit (Pierce Biotechnology, Rockford, IL, USA). Equal amounts of protein (20 μg) were boiled with loading buffer for 10 min. The protein extracts were subjected to electrophoresis on SDS-PAGE gel and then transferred to a nitrocellulose membrane. The membranes were incubated with skim milk (5%) for 1 h at room temperature and then with primary antibodies overnight at 4 °C, followed by incubation with corresponding secondary antibodies. The signals were developed by using an ECL western blot detection kit and visualized on the Carestream molecular imaging system.

### Statistical analysis

The experimental data are expressed as the mean ± standard deviation. One-way analysis of variance (ANOVA) and LSD tests were used to make comparisons among the groups using SPSS 17.0 statistical software. Differences of *P*＜0.05 were considered statistically significant.

## Additional Information

**How to cite this article**: Yang, Y. *et al*. Andrographolide derivative AL-1 ameliorates TNBS-induced colitis in mice: involvement of NF-кB and PPAR-γ signaling pathways. *Sci. Rep.* **6**, 29716; doi: 10.1038/srep29716 (2016).

## Figures and Tables

**Figure 1:**
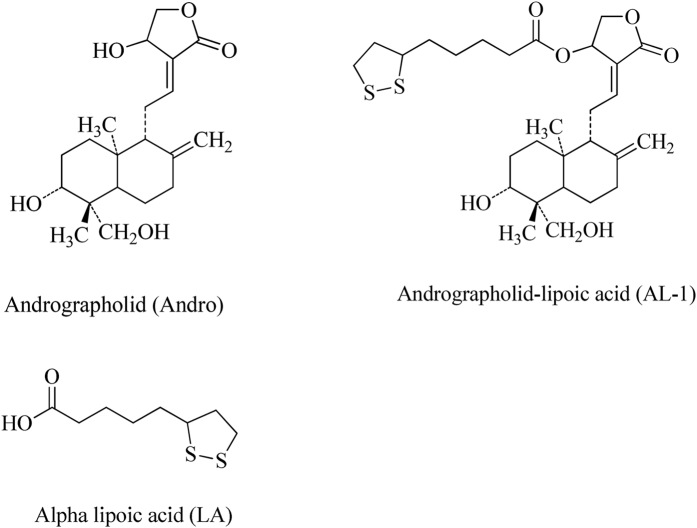
Structures of Andro, LA and AL-1.

**Table 1:** Evaluation of macroscopic scores.

Colon damage	score
No damage	0
Hyperemia without ulcers	1
Hyperemia and wall thickening without ulcers	2
One ulceration site without wall thickening	3
Two or more ulceration sites	4
0.5 cm extension of inflammation or major damage	5
1 cm extension of inflammation or severe damage	6–10

The score was increased by 1 for every 0.5 cm of damage up to a maximal score of 10.

**Table 2:** Evaluation of disease activity index (DAI).

DAI score	Weight loss (%)	Stool consistency	Occult/gross bleeding
0	None	Normal	Normal
1	1–5		
2	5–10	Loose stools	Hemoccult positive
3	10–15		
4	>15	Diarrhea	Gross bleeding

DAI was determined by combining scores of body weight loss, stool consistency and Gross bleeding.

**Table 3:** Evaluation of Colon mucosa damage index.

Colon mucosa damage	Score
Inflammatory cells	
Presence of occasional inflammatory cells in the lamina propria	0
Increased numbers of inflammatory cells in the lamina propria	1
Confluence of inflammatory cells, extending into the submucosa	2
Transmural extension of the infiltrate	3
Tissue damage	
No mucosal damage	0
Discrete lymphoepithelial lesions	1
Surface mucosal erosion or focal ulceration	2
Extensive mucosal damage and extension into deeper structures of the bowel wall	3

The combined histological score ranged from 0 (no changes) to 6 (extensive cell infiltration and tissue damage).
